# Scale-Up of a Heterogeneous Photocatalytic Degradation Using a Photochemical Rotor–Stator Spinning Disk Reactor

**DOI:** 10.1021/acs.oprd.2c00012

**Published:** 2022-03-01

**Authors:** Arnab Chaudhuri, Stefan D. A. Zondag, Jasper H. A. Schuurmans, John van der Schaaf, Timothy Noël

**Affiliations:** † Department of Chemical Engineering and Chemistry, Sustainable Process Engineering, Eindhoven University of Technology (TU/e), 5612 AZ Eindhoven, The Netherlands; ‡ Flow Chemistry Group, van’t Hoff Institute for Molecular Sciences (HIMS), Universiteit van Amsterdam (UvA), 1098 XH Amsterdam, The Netherlands

**Keywords:** heterogeneous photochemistry, flow chemistry, rotor−stator spinning disk reactor, photodegradation, scale-up, solid handling

## Abstract

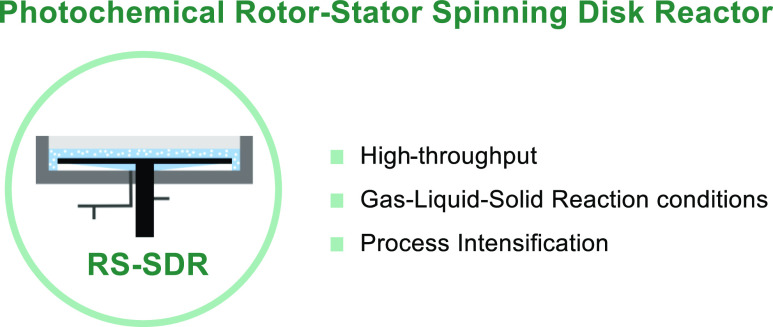

Many chemical reactions contain heterogeneous reagents, products, byproducts, or catalysts, making their transposition from batch to continuous-flow processing challenging. Herein, we report the use of a photochemical rotor–stator spinning disk reactor (pRS-SDR) that can handle and scale solid-containing photochemical reaction conditions in flow. Its ability to handle slurries was showcased for the TiO_2_-mediated aerobic photodegradation of aqueous methylene blue. The use of a fast rotating disk imposes high shear forces on the multiphase reaction mixture, ensuring its homogenization, increasing the mass transfer, and improving the irradiation profile of the reaction mixture. The pRS-SDR performance was also compared to other lab-scale reactors in terms of water treated per reactor volume and light power input.

## Introduction

In recent years, photon-induced transformations have received an upsurge in attention from both academia and industry due to the popularity of photocatalysis.^1^ Advances in LED technologies,^2^ the need for sustainable chemistry^3,4^ and continued efforts to develop and scale up photochemical processes by embracing continuous-flow processing^5−13^ have resulted in a myriad of different reactor designs to bridge the gap between academia and industry. Ranging from the numbering up of micro- and millireactors to the design of completely novel reactor types, the productivities of photochemical reactors have seen tremendous growth over the past years.^14−16^

Despite these important advances, one key issue remains challenging for large-scale continuous-flow photochemical reactions, that is, the clogging problems associated with solid handling, both for the use of solid reactants/catalysts and the generation of solids during operation. Oftentimes, this results in the development of alternative homogeneous systems when shifting from batch to continuous processing, for example, by swapping heterogeneous bases or photocatalysts with homogeneous alternatives.^17^ Apart from the additional efforts required to realize this heterogeneous-to-homogeneous shift, the reaction selectivity can be affected and downstream processing often becomes more cumbersome (e.g., separation and recovery of the homogeneous photocatalyst or byproducts).

The field of solid handling and slurry processing in the continuous manufacturing of chemicals is therefore of major interest to enable the transition from the laboratory scale to industrial fine-chemical production.^18^ Solid particles can be kept in suspension via active or passive solid-management techniques.^19,20^ For example, active solid particle agitation in flow can be achieved by stirring (e.g., continuous stirred-tank reactors^21−23^), ultrasonic irradiation,^24−27^ and oscillatory/pulsation flow,^28^ whereas passive techniques can use static mixing elements or flow-induced agitation (e.g., secondary vortices in multiphase flow regimes).^29,30^ Combinations of active and passive elements have also been reported, such as the merger of static mixing elements and pulsation flow observed in the HANU reactor concept.^28,31−33^

**Figure 1:**
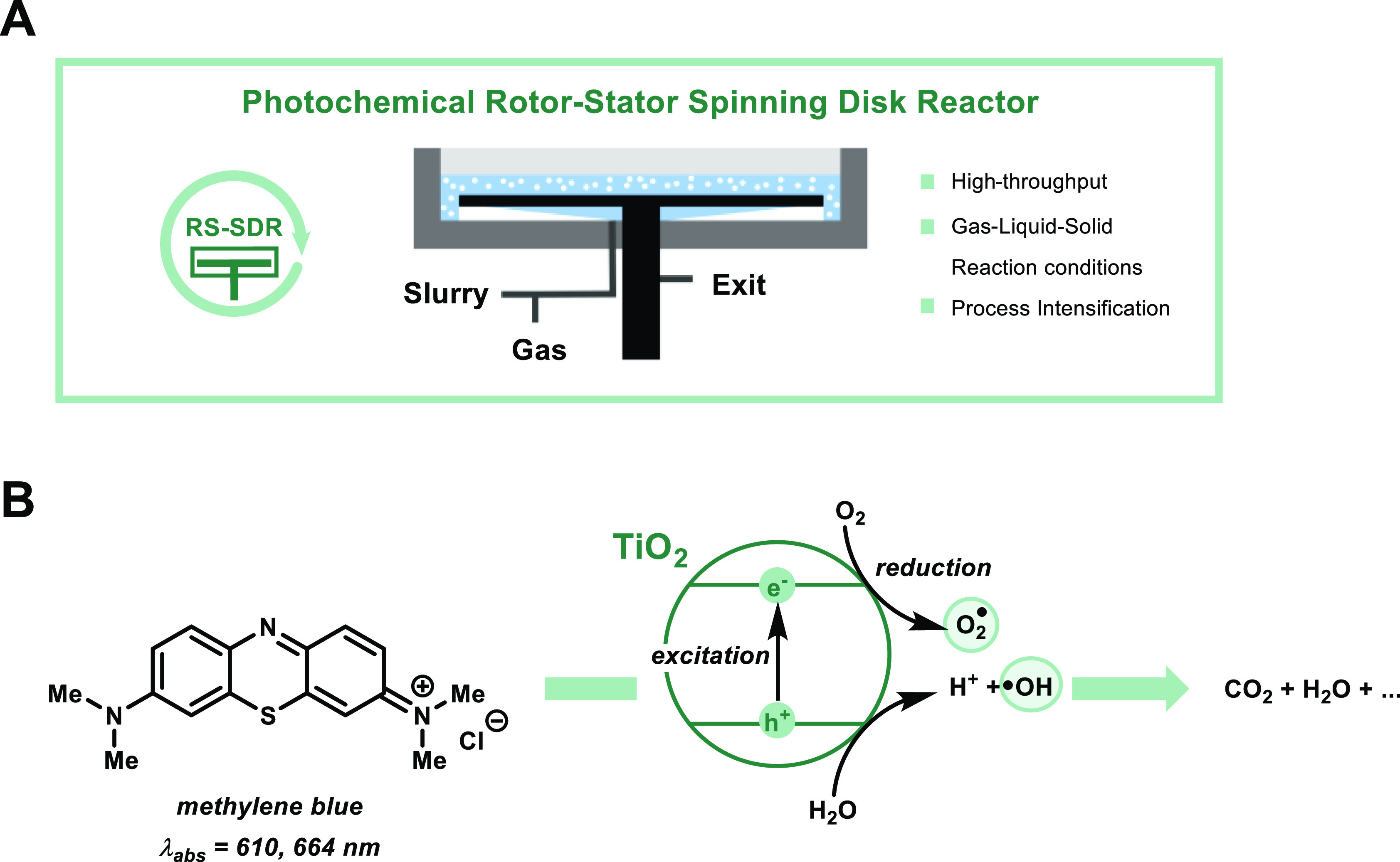
Photocatalytic degradation of MB using TiO_2_ photocatalysis in a pRS-SDR. (A) pRS-SDR enables scale-up of complex heterogeneous photocatalytic reaction conditions. (B) Degradation of MB enabled by titanium dioxide semiconductor photocatalysis.

Another technology which holds promise for solid/slurry handling in flow is the rotor–stator spinning disk reactor (RS-SDR) (Figure 1A). This reactor type can generate a high degree of shear and turbulence in the reaction mixture from the rotation of a disk (∼130 mm in diameter) in a narrow rotor–stator gap, either using a thin film or in dispersed operation mode. As a consequence, high mass^34−40^ and heat transfer^41,42^ rates have been reported for the spinning disk reactor system, which in turn have been used to intensify a number of chemical reactions/processes.^43−47^ An application of this reactor, adapted to enable homogeneous irradiation of the dispersed reaction mixture, has been recently published by our groups for an intensified photochemical gas–liquid process.^48^ In this paper, we demonstrate that the photochemical RS-SDR (pRS-SDR) in its dispersed operation mode can be exploited for the handling of solid-containing heterogeneous photochemical systems.

As a suitable benchmark reaction, we selected the photocatalytic degradation of organic dyes, which are problematic to remove from wastewater effluents using conventional purification methods (Figure 1B).^49−51^ For this application, semiconductors are often used as recoverable heterogeneous photocatalysts; a typical example being titanium dioxide (TiO_2_) due to its chemical stability, nontoxic nature, low cost, and high photocatalytic activity.^52,53^ The electron–hole pairs generated upon the excitation of TiO_2_ can produce reactive radicals (so-called reactive oxygen species, Figure 1B), which nonselectively oxidize the organic pollutants, such as methylene blue (MB), to carbon dioxide, water, and various mineralization products.^50,54−56^ Interestingly, the photodegradation of organic dyes using TiO_2_ has been extensively researched over the years and has served as a benchmark reaction for novel reactor designs, ranging from laboratory to intermediate scales and from batch to continuous-flow operation modes.^57−65^ An additional advantage of this transformation is the possibility of easily assessing the remaining concentration of MB using conventional UV–vis spectroscopical tools. In this work, we report on the ability of the pRS-SDR to process complex aqueous gas–liquid–solid reaction streams without clogging and demonstrate that high productivity rates can be obtained for the TiO_2_-mediated aerobic photodegradation of aqueous MB. We have also evaluated this photodegradation experimentally in a batch reactor and compared the pRS-SDR and batch reactor with other reactor types in the literature.

## Experimental Section

### General Information

Solutions with the desired concentration of MB (10 ppm) were made using demineralized water as the solvent, and a certain amount of TiO_2_ (Evonik Aeroxide P25, 21 nm) was added. Gaseous O_2_ was supplied with a mass flow controller (Bronkhorst EL-FLOW) and set to the desired flow rate. The samples taken from the reaction mixture were first diluted with ethanol before the solid particles were filtered using a syringe filter (CHROMAFIL Xtra PTFE 0.2 μm). The use of ethanol was also necessary to remove any adsorbed MB on the TiO_2_ into the analyte. Analysis of the samples was done using a spectrophotometer (UV-2501PC). A calibration curve was made at λ = 657 nm (see the Supporting Information, Figures S1 and S2) and was used to obtain the conversion of MB.

### Batch Setup

The batch setup employed in our work is schematically shown in the Supporting Information (Figure S4). Inside a 3D-printed cylindrical vessel, an LED strip was attached (365 nm UV, 60 W maximum input power, see the Supporting Information, Figure S3, for emission spectrum). This strip was connected to an external power supply, which could regulate its power input. To keep a constant power per volume during the experiments, the power was adjusted after taking a sample to account for the decrease in volume. The vial (Pyrex, 7.5 mL) containing the reaction mixture (4 mL) was placed inside the photochemical vessel through the lid, and oxygen was supplied via a needle placed in the reaction mixture. Mixing occurred via a magnetic stirrer at a fixed position for all experiments. The reactor was air-cooled to maintain the reaction mixture at room temperature.

### pRS-SDR Setup

The construction of the reactor setup for visible-light photochemistry has been previously reported.^48^ The relevant internal dimensions of the reactor are given here for completeness. The rotor has a diameter of 130 mm, the distance between the rotor and stators is kept to 2 mm, and the total volume of the reactor is 64 mL, where the irradiated volume makes up 27 mL. However, due to the use of UV-A light, the setup was covered to shield the operator from harmful irradiation. On top of the reactor, the cover containing the light source was placed (Figure 6). The used light source was an LED floodlight placed on a mount (365 nm UV, 175 W maximum input power, see the Supporting Information, Figure S3, for the emission spectrum). The cover was constructed by the Equipment and Prototype Center at the TU Eindhoven and was made of stainless steel and insulation materials. The top part also holds a fan, allowing for extra cooling, in addition to the heat sink of the light source. The electrical system is supplied with a fuse, so the system automatically shuts down in case of overheating.

The suspension was fed with a peristaltic pump (Masterflex Ismatec) and combined with the O_2_ flow before entering the reactor via a T-mixer (reactor setup schematically shown in the Supporting Information, Figure S5). The vessel containing the suspension was covered from light and stirred continuously. The temperature was monitored at the outlet of the reactor using a thermocouple, and the temperature rise was typically no higher than 8 °C at steady state. For each data point, two samples were taken after at least three residence times to ensure a steady state.

## Results and Discussion

To investigate the photodegradation of aqueous MB, experiments were first performed using a small-scale batch reactor. The reaction with MB is particularly interesting since the aqueous solution gradually loses its bright blue color upon degradation and can be followed spectroscopically.^66^ The presence of an oxidant (e.g., oxygen and hydrogen peroxide) that reacts with the excited electron in the conduction band of TiO_2_ is of great importance since this can prevent the electron–hole recombination of the excited photocatalyst.^67^ In this study, oxygen (O_2_) was chosen, resulting in a triphasic gas–liquid–solid reaction system.

### Photodegradation in Batch

**Figure 2:**
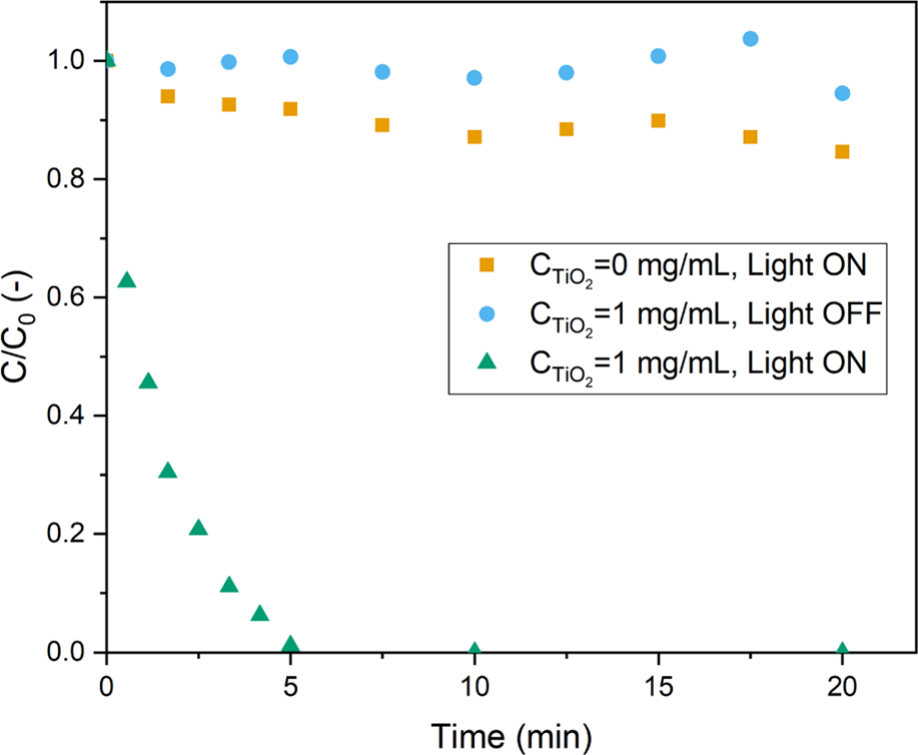
Normalized concentration of MB vs time. The behavior in absence of UV irradiation or the catalyst is compared to the presence of both ([light power input 52 W (13 W/mL), 10 ppm MB, and 4.0 mL/min O_2_ bubbling].

Initially, control experiments were carried out in the absence of TiO_2_, but with exposure to UV irradiation at the highest input power, to investigate the effect and significance of the direct photolysis pathway (Figure 2).^68^ This experiment does result in some minor MB degradation, but its extent is negligible relative to the photocatalytic pathway, where full conversion is observed within 5 min of reaction time. Additionally, another control experiment without light irradiation but with the photocatalyst was conducted. In the absence of light irradiation, the adsorption of MB on the photocatalyst is noticeable but does not result in any MB degradation.

Next, the dependencies of the reaction rate on light intensity, TiO_2_ concentration, and O_2_ bubbling rate were investigated. A first-order reaction rate in the concentration of MB was found to approximate the overall photocatalytic system well (eq 1), where the apparent overall reaction rate constant (*k*) encompasses all other factors influencing the reaction rate (e.g., mass transfer and light attenuation).
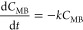
1

**Figure 3:**
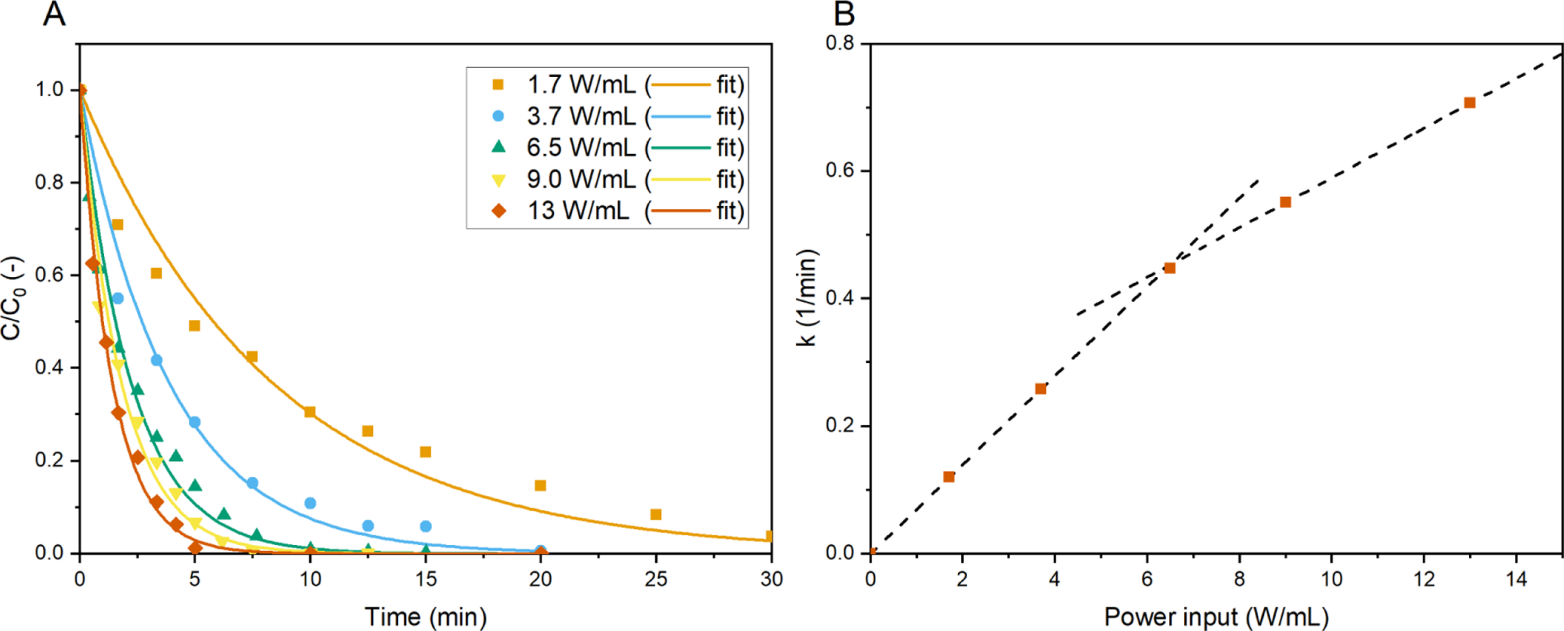
Normalized concentration of MB (A) apparent overall reaction constants (B) vs the time for different UV LED power inputs per volume with their fitted first-order concentration–time curves (10 ppm MB, *C*_TiO_2__ = 1.0 mg/mL, and 4.0 mL/min O_2_ bubbling).

We commenced with varying the power input of the UV LED strip using an external power supply to set the effective light intensity irradiating the sample. In Figure 3, the normalized MB concentrations for various power inputs per volume in time are given. Higher light intensities clearly enhance the reaction rate, where the apparent reaction rate constant for 13 W/mL (0.71 1/min) is almost sixfold compared to the one at 1.7 W/mL (0.12 1/min). However, the relation of the apparent reaction rate constant to the input power was found only to be linear for the additional intensity at lower power inputs (for <6.5 W/mL, see Figure 3B). This indicates a possible shift away from a fully photon-limited system, causing other factors to become more limiting.^6^ Extension of the operating regime where higher light intensities result in faster degradation could possibly be achieved by intensifying mass transfer or by operating at increased concentration of the light-absorbing species, that is, the photocatalyst TiO_2_.

**Figure 4:**
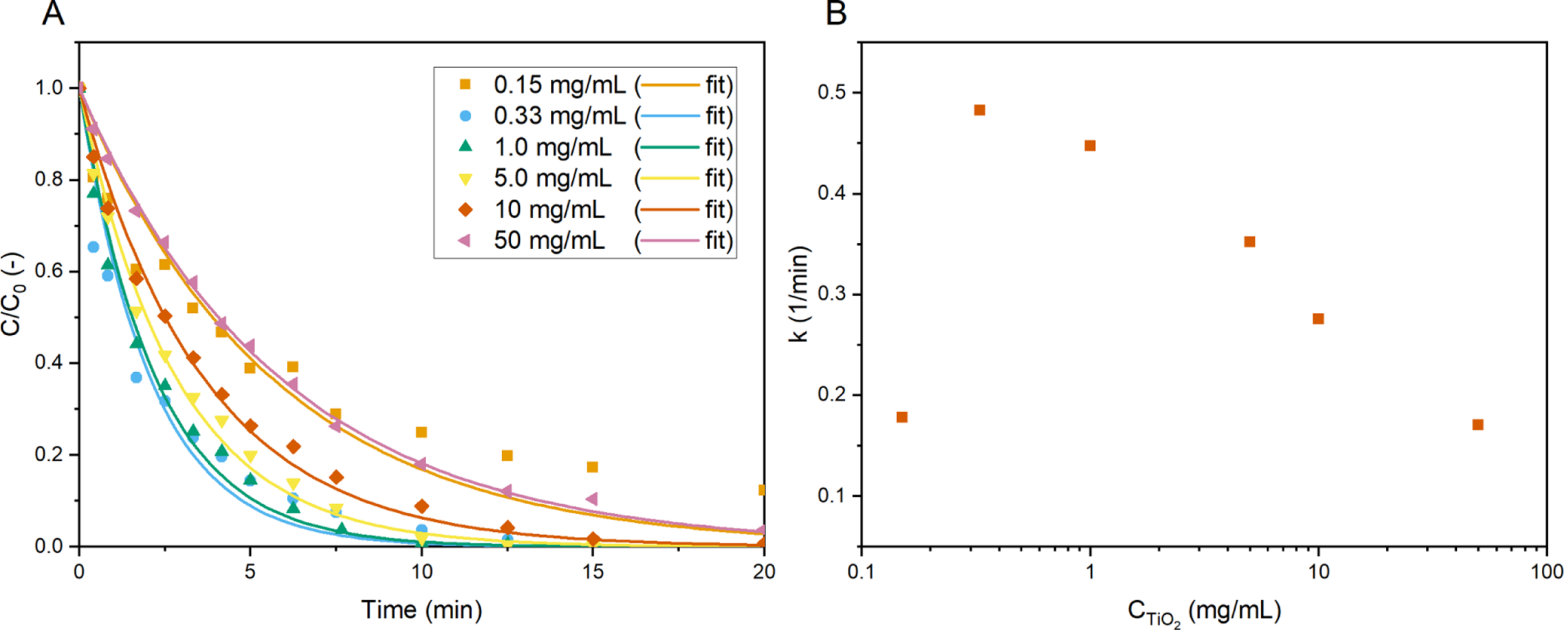
Normalized concentration of MB (A) and the apparent overall reaction constants (B) vs the time for varying catalyst concentrations [light input power 26 W (6.5 W/mL), 10 ppm MB, and 4.0 mL/min O_2_ bubbling].

Next, the photocatalyst concentration was varied while maintaining the irradiation at a power input of 6.5 W/mL. This was done for the ease of comparison with the continuous-flow setup (see the Supporting Information), where this is the maximum power input with the current configuration. Increasing the catalyst concentration increases the concentration of the light-absorbing species, allowing more photons to be absorbed. However, a too high concentration can also result in a nonhomogeneous irradiation and lead to additional irradiation losses caused by light scattering. Therefore, an optimal catalyst concentration was expected and found at 0.33 mg/mL (*k* = 0.48 1/min, see Figure 4). Notably, the previously used value of 1.0 mg/mL yielded a comparable rate constant (*k* = 0.45 1/min), while for higher and lower catalyst concentrations, the rate constant significantly dropped. Importantly, the suspension remained stable at relatively long time intervals: even at the highest catalyst concentration, no sedimentation of particles was observed at the bottom of the reactor.

**Figure 5:**
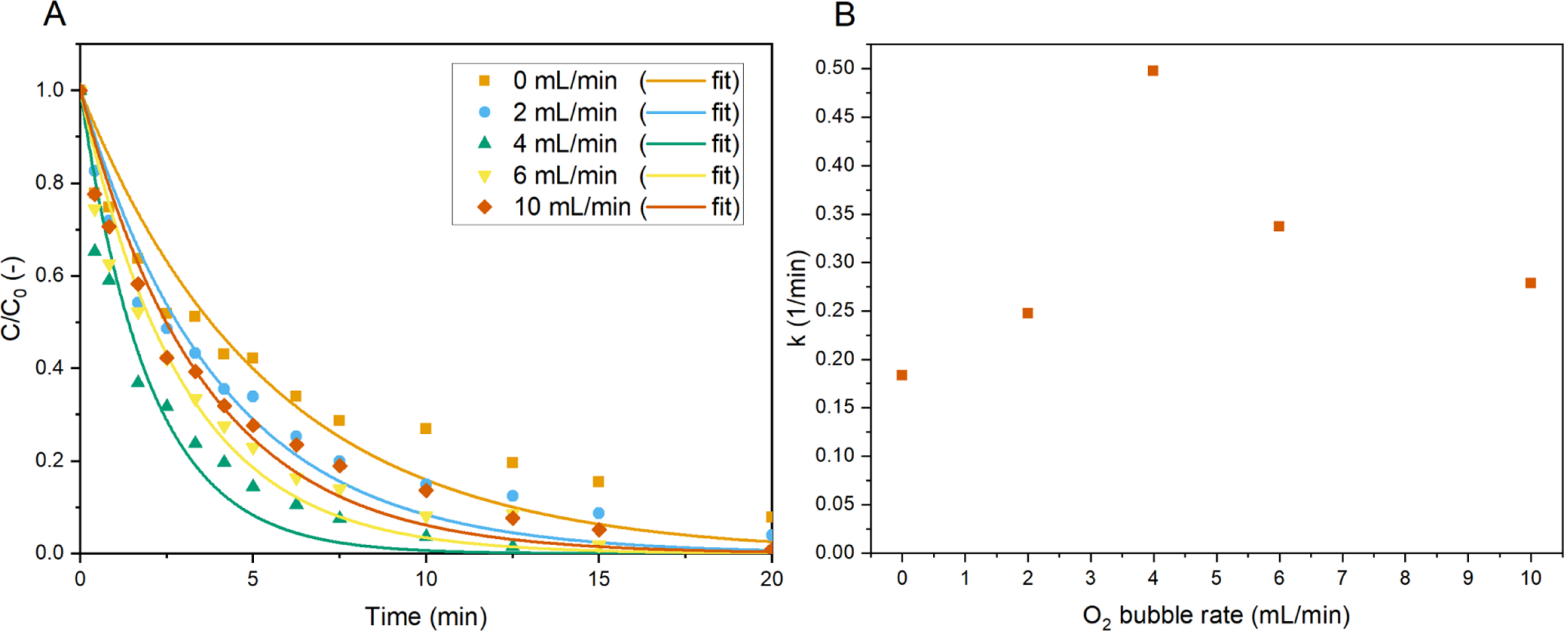
Normalized concentration of MB (A) and the apparent overall reaction constants (B) vs the time for different O_2_ bubbling flow rates [light input power 26 W (6.5 W/mL), 10 ppm MB, and *C*_TiO_2__ = 0.33 mg/mL].

Evaluation of the gas–liquid mass transfer effects in batch was done by varying the oxygen flow rate bubbled through the reaction mixture. These experiments were again conducted at a power input of 6.5 W/mL and the experimentally determined optimal catalyst concentration of 0.33 mg/mL. The flow rate of oxygen was varied from 0 to 10 mL/min, where at 0 mL/min, the headspace above the reaction mixture was O_2_. The batch system could not be described well for flow rates other than 4 mL/min by the apparent first-order reaction kinetics, but the fitted curves are still shown and used to get an approximation of the reaction constants (Figure 5). The data shows that the oxygen flow rate influences the kinetic rate in a complicated way. At zero flow, it is evident that the oxygen saturation level of the mixture is sufficient to convert all the MB, although oxygen mass transfer may become the rate-limiting step at high conversions. Adding a bubble flow maintains a high-enough oxygen concentration to have MB as the rate-limiting reactant. The optimal flow rate was found to be 4.0 mL/min. At lower flow rates, the gas-to-liquid (G/L) mass transfer could be limiting the reaction rate, where the surface available for G/L mass transfer is not sufficient. It should also be noted that the gas bubbling also contributes to additional mixing of the reaction mixture. At flow rates higher than 4.0 mL/min, the formed bubbles appear larger in size, resulting in shorter contact times (due to higher bubble rise velocities) and could increase irradiation losses caused by scattering and transmission.

### Photodegradation in the pRS-SDR

**Figure 6:**
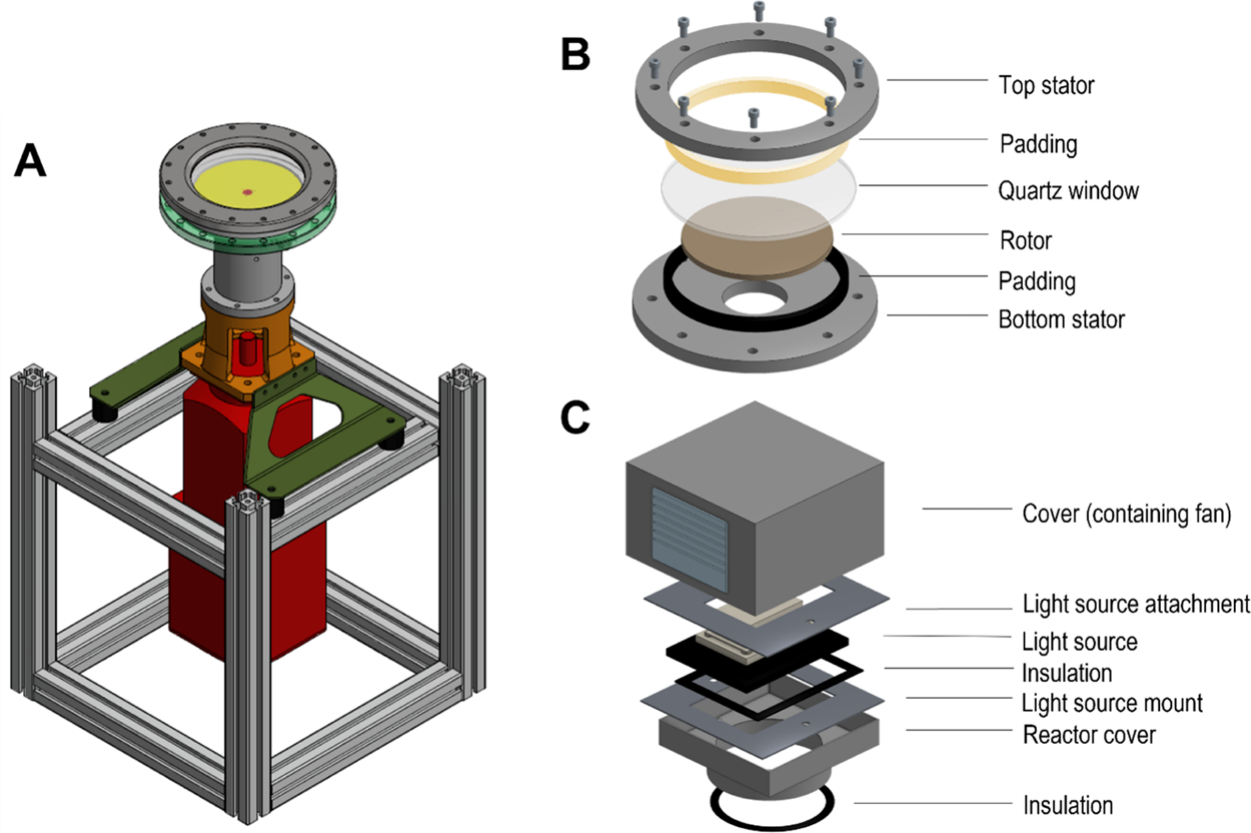
Schematic representation of the pRS-SDR. (A) Schematic of a fully assembled reactor without the cover. (B) Exploded view of the reactor. (C) Exploded view of the cover for the UV light source.

After investigation of the photodegradation of MB in batch, our research focus shifted toward the use of the pRS-SDR (schematically shown in Figure 6).

For multiphase continuous-flow processes, the liquid volume in the reactor is determined by the G/L ratio of the feed^48^ and the solid volume fraction. The residence time in the pRS-SDR is relatively independent of the rotation as the differences in holdup are typically small for varying rotation speeds.^34,35,48,69^ The rotation speed directly affects the mixing and mass transfer, effectively decoupling the mass transfer from the flow rate or residence time, which distinguishes spinning disk reactors from nonagitated flow reactors. A relatively high catalyst concentration of 10 mg/mL (∼1.0 w % of the suspension) was chosen for the standard conditions in order to illustrate the ability of the pRS-SDR to handle high solid concentrations. The volumetric G/L ratio was generally kept at 1:1 to ensure an excess of O_2_.

**Figure 7:**
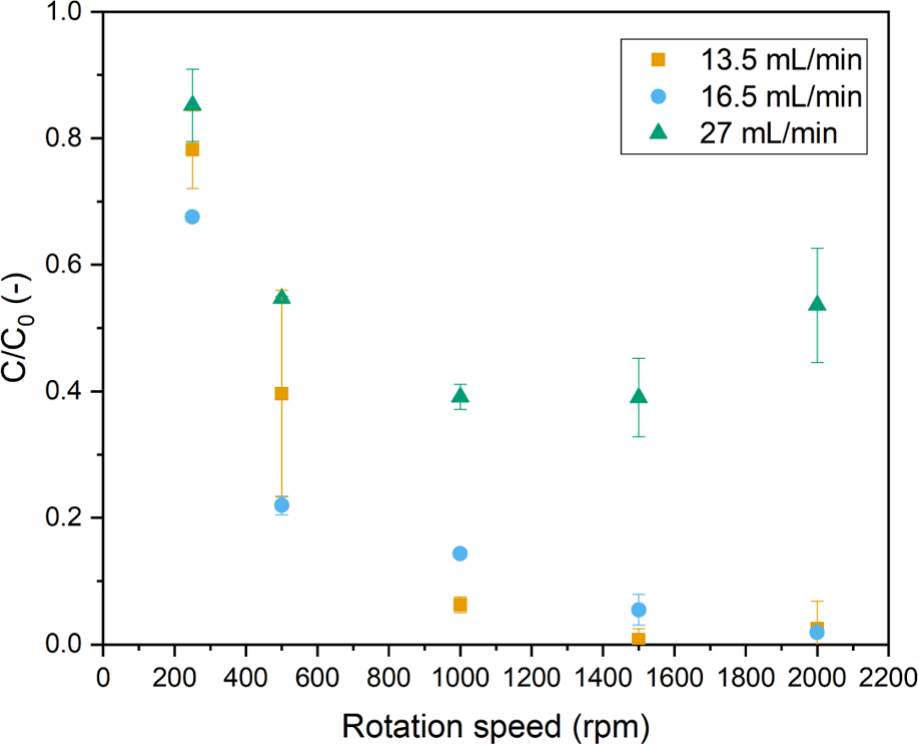
Normalized concentration of MB vs rotation speed for different flow rates, with a G/L ratio of 1:1 [light input power 175 W (6.5 W/mL), 10 ppm MB, and *C*_TiO_2__ = 10 mg/mL].

Initially, the liquid flow rate (Φ_V,L_) was varied while keeping the G/L ratio constant at 1:1 (Figure 7). The degradation of MB was enhanced drastically by increasing the rotation speed. This may be caused by an increase in liquid–solid and gas–liquid mass transfer, by increasing the dispersion of gas and solids in the liquid, by breaking up solid agglomerations, and/or by the reduction of the bubble size, allowing for more efficient gas–liquid mass transfer. Notably, the mixing efficiency reduces the light-penetration limitations we observed in the batch reactor as the reactants and catalyst are continuously replenished at the high-irradiation zones of the reactor, that is, at the quartz window. We have also estimated the resulting average *k* value (please see the Supporting Information for calculation details) in the pRS-SDR at 250 rpm (*k* = 0.061 1/min) and at 1500 rpm (*k* = 70 1/min) for the flow rate of 16.5 mL/min. This analysis clearly indicates the improvement in reaction rate due to an improvement in mixing. For comparison, the highest *k* value observed at the same light intensity (6.5 W/mL) in batch was *k* = 0.5 1/min. Of course, as Figure 7 also indicates, increasing the liquid flow rate and effectively decreasing the liquid residence time, leads to an increase in concentration of the remaining MB at the reactor outlet.

**Figure 8:**
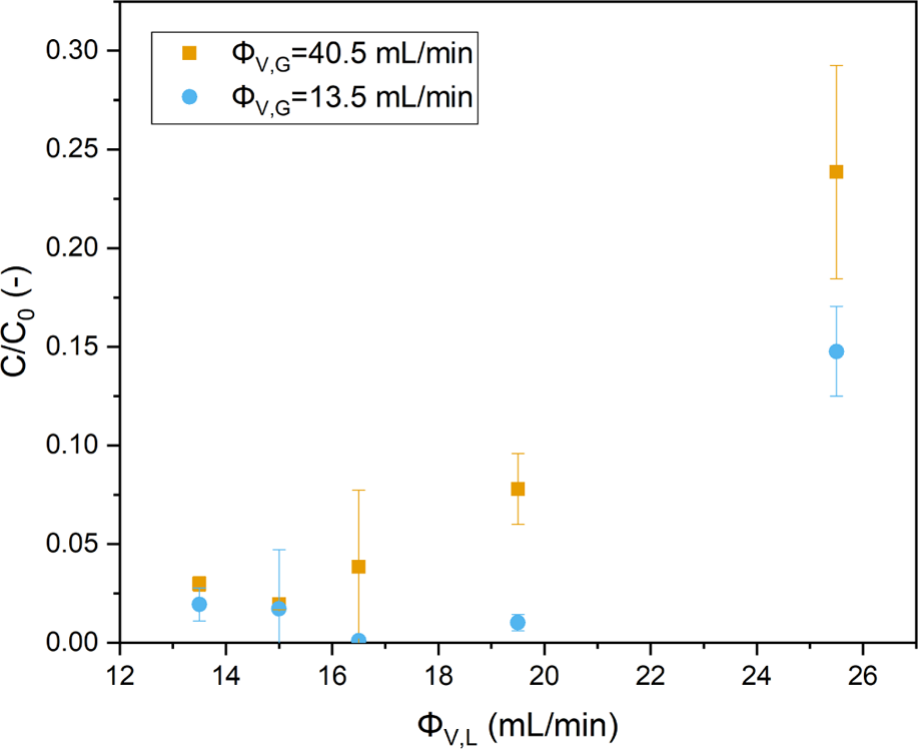
Normalized concentration of MB vs the liquid flow rate for two different constant gas flow rates at 2000 rpm [light input power 175 W (6.5 W/mL), 10 ppm MB, and *C*_TiO_2__ = 10 mg/mL].

Next, the G/L ratio was increased to 3:1 and this situation was compared with a 1:1 ratio at a constant liquid flow rate (13.5 mL/min, see the Supporting Information, Figure S10). With a 3:1 G/L ratio, higher conversions could be reached at lower rotation speeds compared to a 1:1 G/L, suggesting the existence of gas–liquid mass transfer limitation of oxygen. At higher rotation speeds (∼1000 rpm), this effect diminished and both G/L ratios reached full degradation of MB. Further investigation of the G/L ratio was performed by varying Φ_V,L_ at the highest rotation speed of 2000 rpm to ensure the best possible mixing and mass transfer. Two sets of experiments were performed, with the gas flow rates (Φ_V,G_) being kept constant at two separate values (Figure 8). For the constant gas flow rate of 13.5 mL/min, the maximum observed liquid flow rate that resulted in >90% MB degradation was found to be 19.5 mL/min. This yielded the best reactor performance thus far. We have used this value for the comparison between various reactor designs in the next section. No significantly different behavior is found at the higher gas flow rate, even though the performance at especially the higher liquid flow rates starts to worsen slightly. This might be attributed to an increase in the gas holdup, effectively lowering the liquid residence time.

**Figure 9:**
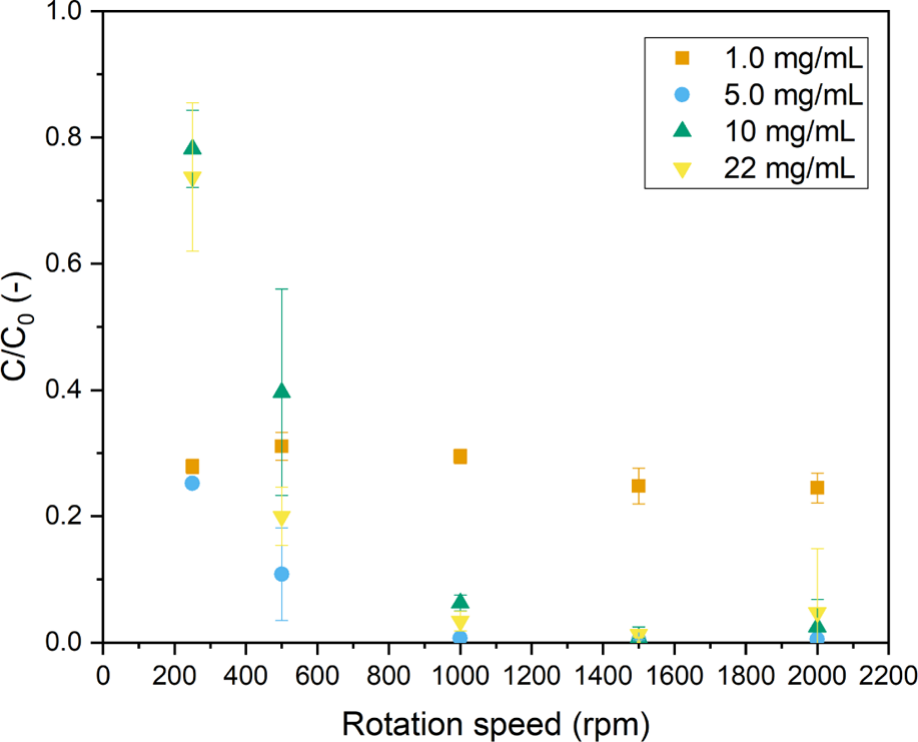
Normalized concentration of MB vs rotation speed for different catalyst concentrations/loadings [light input power 175 W (6.5 W/mL), Φ_V,L_ = 13.5 mL/min, G/L ratio 1:1, 10 ppm MB.

Increasing the TiO_2_ loading can increase the total amount of active sites and thus the adsorption of substrates. However, a higher TiO_2_ loading results simultaneously in increased light scattering and light attenuation, limiting the maximum observed reaction rate. Several catalyst concentrations have been screened at a liquid flow rate of 13.5 mL/min and a G/L ratio of 1:1. The results in Figure 9 show that for the investigated concentrations, only in the case of 1.0 mg/mL, full MB degradation cannot be reached, even at elevated rotation speeds. For all other catalyst loadings, increasing the rotation speed directly translates into higher conversions. This implies that for the higher catalyst loadings, the higher rotation speeds can overcome light penetration limitations (more opaque reaction solution due to higher heterogeneity). At higher rotation speeds, it can be expected that the solid catalysts are better dispersed in solution, leading to a higher fraction of surface area being available for the reaction. These factors in combination with improvements in mass transfer (solid–liquid and gas–liquid) are the most likely cause for the enhancements observed within the pRS-SDR.

**Figure 10:**
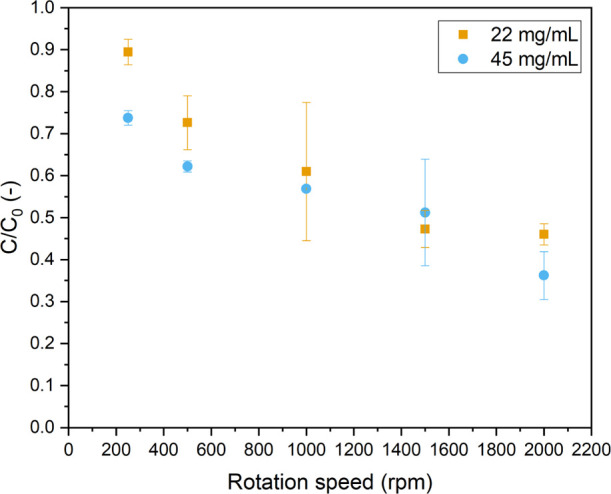
Normalized concentration of MB vs rotation speed for higher catalyst concentrations to test the limitations of the system [light input power 175 W (6.5 W/mL), Φ_V,L_ = 27 mL/min, G/L ratio 1:1, 10 ppm MB.

Since almost all catalyst concentrations in Figure 9 reach full conversion around 1000 rpm, the liquid residence time was further decreased by doubling the liquid flow rate to 27.0 mL/min (while keeping the G/L ratio constant at 1:1). Under these reaction conditions, full conversion will not be reached, which would allow us to identify practical limitations at higher catalyst loadings. Figure 10 shows indeed that full degradation of MB was not reached. However, the conversion improves consistently at increasing rotation speeds, even for catalyst concentrations as high as 45 mg/mL. Notably, no issues with the handling of this concentrated slurry were encountered during reactor operation (see the Supporting Information).

### Reactor Comparison

In this section, the batch reactor and the pRS-SDR are compared to other reactor types for the TiO_2_-enabled photodegradation of MB. Table 1 gives an overview of the reactor types, conditions, and performance of these reactors. Our work is compared to two reactor types using an immobilized photocatalyst and two reactor types operated under slurry conditions. The systems are benchmarked in two different ways to calculate the productivity. The first one is the amount of polluted water treated per unit of time per reactor volume, corrected for the power input of the light source (relative productivity I). Since this number can favor reactors with a very low reactor volume and a low-power light source, this is considered not entirely suitable for scale-up investigations. Therefore, the reactors are also evaluated in terms of the amount of water treated per power of the light source and per unit of time (relative productivity II).

**Table 1:** Comparison of Some Reactor Types Used for the Photodegradation of MB Using TiO_2_^a^

reactor type	TiO_2_ usage	MB (ppm)	light input power and wavelength	reactor volume (mL)	reaction time (min)	relative productivity I^b^	relative productivity II^c^	source
thin-film spinning disk	immobilized	10	20 W	∼0.09	>167	0.073	2.54 × 10^–4^	(71)
			254 nm					
								
annular reactor	immobilized	8	20 W	201	120	0.10	0.75	(72)
			254 nm					
								
batch reactor	suspension *C*_TiO2_ = 2.5 mg/mL	2.1	125 W	20	120	0.016	0.25	(68)
			340 nm					
								
CPC reactor	suspension *C*_TiO2_ = 0.4 mg/mL	10	60 W	600	>180	0.022	0.50	(73)
			365 nm					
								
batch reactor (this work)	suspension *C*_TiO_2__ = 0.33 mg/mL	10	26 W	4	10	0.74	0.14	
			365 nm					
								
pRS-SDR (this work)	suspension *C*_TiO_2__ = 10 mg/mL	10	175 W	27	<1.4^d^	1	1	
			365 nm					

a The productivities do not take into account the initial MB concentrations of the treated water.

b Water flow treated (mL/s) per reactor volume (mL) per light power input (W). Normalized to the pRS-SDR: 6.9 × 10^–5^ 1/(s·W).

c Water flow treated (mL/s) per light power input (W). Normalized to the pRS-SDR: 1.9 × 10^–3^ mL/(s·W).

d This is the liquid residence time at an assumed gas holdup of 0%, making this the maximum possible residence time. Gas holdups of <20% are expected.^48^

The pRS-SDR has the best performance for both comparison methods. It should be noted that the immobilized designs make use of a light source of 254 nm, which is more expensive and increases the effect of direct photolysis. The compound parabolic collector (CPC) reactor displays good performance at low catalyst concentrations. However, handling higher concentrations led to a decrease in the reactor performance, making this reactor type less suitable for subsequent process intensification and scale-up. A disadvantage of the pRS-SDR is that the rotor-induced mixing will lead to additional energy losses, which have not been accounted for in Table 1. This energy dissipation will increase with increasing rotation speed, where the amount of energy dissipated is estimated to be 37 W at 2000 rpm (accounting for <20% of the total power consumption).^70^ Therefore, it is vital to optimize the reaction system so that the excess energy dissipated by the rotor translates to an increase in productivity.

The pRS-SDR shows excellent performance for the photodegradation and, most importantly, encountered no issues with solid handling. For the investigated catalyst concentrations, light penetration issues can actively be overcome by tuning the rotation speed of the rotor. The ability to increase mass transfer and obtain excellent mixing would also allow for process intensification, where concentrations of the reactants and catalyst can be increased together with the power of the light source. It should also be noted that the reactor system has not yet been fully optimized and, thus, the performance of the reactor can possibly be increased, for example, by using higher-intensity light sources.

## Conclusions

Herein, the pRS-SDR was validated for continuous-flow heterogeneous photochemistry using the TiO_2_-mediated aerobic photodegradation of MB as a benchmark transformation. This high-shear photochemical reactor is uniquely suited to handle such complex solid-containing reaction mixtures and did not display any sign of reactor clogging, a problem often associated with other continuous-flow capillary reactors. The use of a fast rotating disk ensured homogenization of the reaction mixture, increased the mass transfer, and improved the irradiation profile of the reaction mixture. We anticipate that the pRS-SDR will aid in the transition from batch to continuous-flow operation in the pharmaceutical and agrochemical industry by facilitating the scale-up of challenging multiphase synthetic transformations.

## Supporting Information Available

Absorption and light source spectra, schematic batch and pRS-SDR reactor setups, overall reaction kinetics fitting methods, additional pRS-SDR behavior characteristics (e.g., extent of reactor fouling), and qualitative image analysis (PDF)
