# Supplementary material for: Scale-Up of a Heterogeneous Photocatalytic Degradation Using a Photochemical Rotor–Stator Spinning Disk Reactor
Source: Org Process Res Dev. 2022 Mar 1;26(4):1279–88. doi: 10.1021/acs.oprd.2c00012 (PMC9017180; doi:10.1021/acs.oprd.2c00012)
Supplement: Supplementary file 1 — op2c00012_si_001.pdf [file op2c00012_si_001.pdf]

Supporting Information: Scale-up of a heterogeneous photocatalytic degradation using a photochemical Rotor-Stator Spinning Disk Reactor

Arnab Chaudhuri[1†], Stefan D. A. Zondag[2†], Jasper Schuurmans[1], John van der Schaaf[1], Timothy Noël[2]*,*

[1]Department of Chemical Engineering and Chemistry, Sustainable Process Engineering, Eindhoven University of Technology (TU/e), 5612 AZ Eindhoven, The Netherlands.

[2]Flow Chemistry Group, van 't Hoff Institute for Molecular Sciences (HIMS), Universiteit van Amsterdam (UvA), 1098 XH Amsterdam, The Netherlands.

[†]*Equal Contribution*

[*]*Corresponding Author e-mail: j.vanderschaaf@tue.nl (J. van der Schaaf), t.noel@uva.nl (T. Noël)*

Contents

1. MB absorption, calibration, and light sources 2
2. Reactor setups 4
3. Batch results 5
4. Flow results 8
5. High Speed Imaging of the pRS-SDR 10
6. References 13

1. **MB absorption, calibration, and light sources**

The absorption spectrum of methylene blue in the ethanol-diluted aqueous sample is shown in **Figure S1** with a maximum absorption peak at 657 nm. The calibration curve used to relate the sample's absorbance to their concentration is shown (and the equation given in the caption of) **Figure S2**.

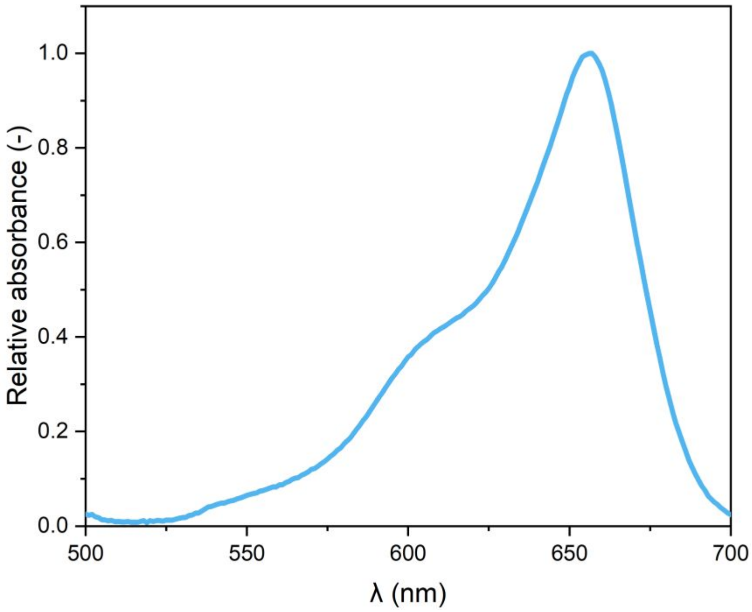

Figure S1. Absorption spectrum of methylene blue relative to the maximum absorbance.

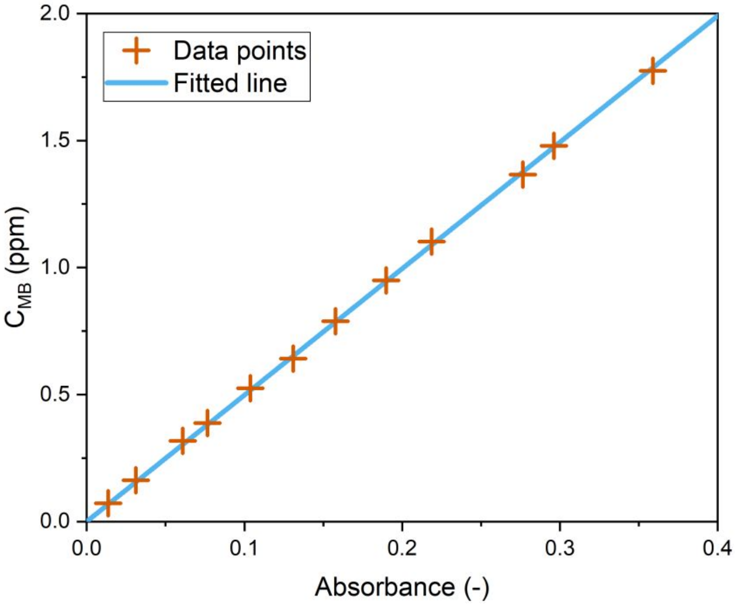

Figure S2. Calibration curve for the determination of the MB concentration. Absorbance measured at 657 nm. (y=4.9775x, R^2=0.9999)

The wavelength-dependent relative intensities (emission spectra) of the light sources used in batch and for the pRS-SDR are shown in **Figure S3**.

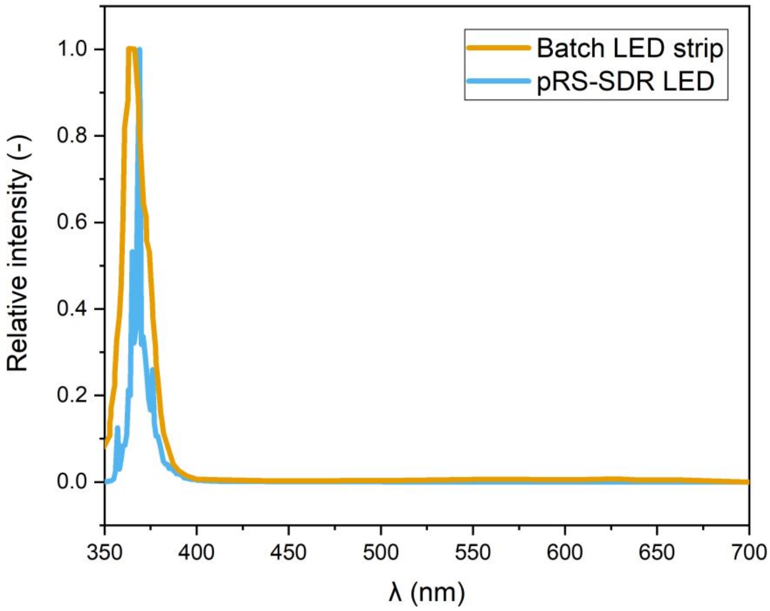

Figure S3. Wavelength-dependent relative intensities of the light sources used for the batch experiments (60 W maximum input power, 2.36% efficiency) and the pRS-SDR (175 W maximum input power, 2.39% efficiency).

2. **Reactor setups**

Schematic representations of the batch reactor (**Figure S4**) and the pRS-SDR (**Figure S5**) are shown here. In the batch reactor the O_2 is bubbled through a needle fed by a mass flow controller. For the pRS-SDR the O_2 is also provided by the same mass flow controller. A picture of the flow reactor setup is also provided. The specific irradiated area was 5.2 cm^2/mL for the batch reactor and 5.22 cm^2/mL for the pRS-SDR and can be used to convert the light intensities given in W/mL to W/cm^2 by dividing by this specific irradiated area.

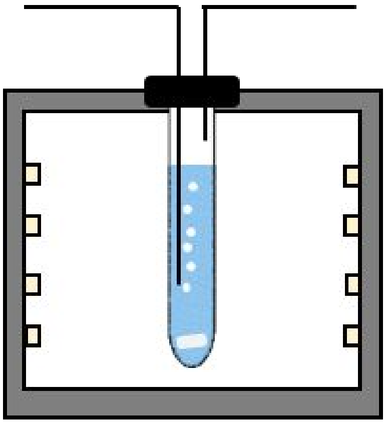

Figure S4. Schematic representation of the batch reactor setup. The LED strip provides irradiation from the cylindrical 3D-printed vial-holder. The 7.5 mL vial is filled with 4 mL reaction mixture and stirred with a magnetic stirrer.

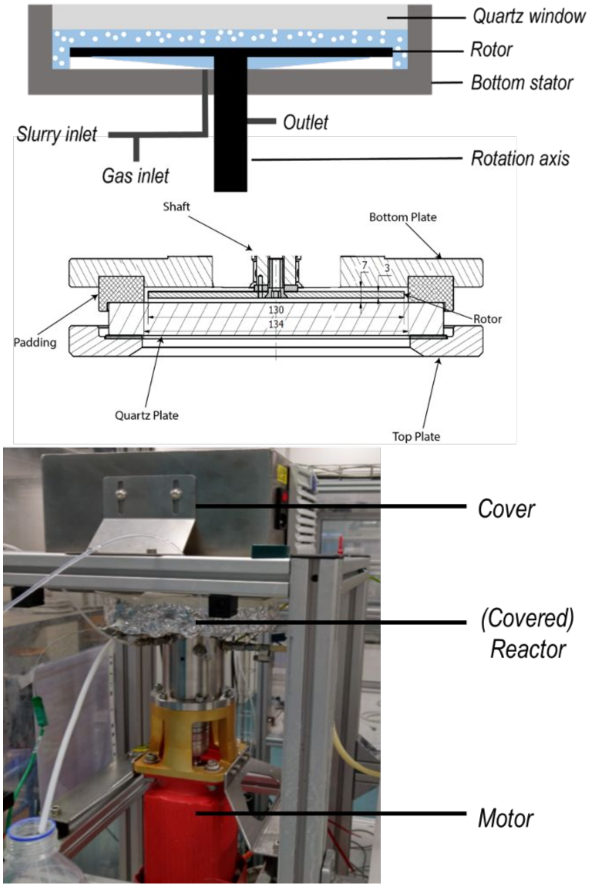

Figure S5. Top) Schematic representation of the pRS-SDR setup. Middle) Technical design drawing of the reactor, including the rotor shaft, quartz window and stator assembly including the basic dimensions in mm. Bottom) Picture of the assembled reactor. The reactor is irradiated through the quartz window. The internal volume is 64 mL, but the irradiated volume is 27 mL.

3. **Batch results**

The reaction rate constant, k, can be obtained from plotting **Eq. S2** (derived from **Eq. S1**) as $-k$ is the slope of the plotted graph (**Figure S6**, where linear fitting is used).

$$\frac{dC_{MB}}{dt} = -kC_{MB} \qquad \textbf{Eq. S1}$$

$$\ln\left(\frac{C_{MB}}{C_{MB,0}}\right) = \ln(1-X) = -kt \qquad \textbf{Eq. S2}$$

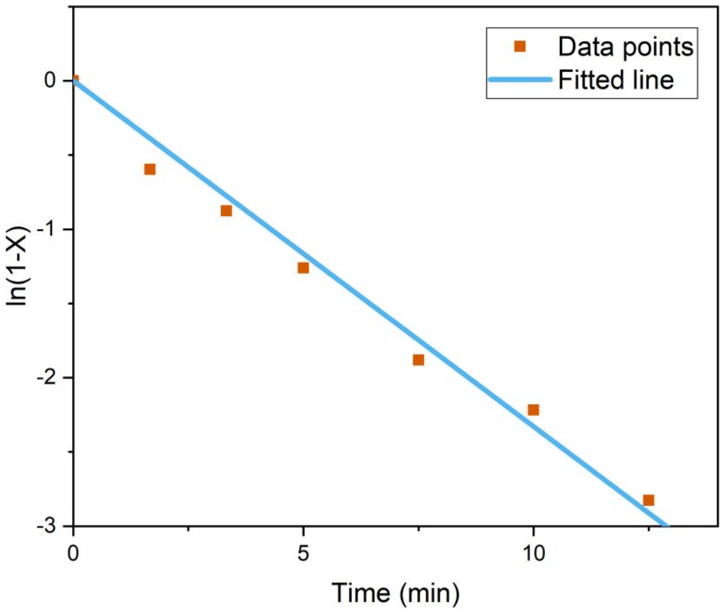

Figure S6. Linear fit of first order reaction of the MB degradation in batch. Light power input 60 W (13 W/mL), 10 ppm MB, $C_{TiO_2} = 1.0$ mg/mL, 4.0 mL/min O_2 bubbling. (y=-0.2329x, R^2=0.9948)

Additionally, apart from the linear, also a non-linear least-squares analysis was performed (**Figure S7**). The fitted apparent first order reaction constant for the linear least-squares analysis was 0.2329 min^{-1}. For the nonlinear analysis a value of 0.2580 min^{-1} was found. The root mean square error (RMSE) for the nonlinear analysis was found to be $3.8 \cdot 10^{-2}$, whereas the RMSE of the linear analysis was equal to $4.7 \cdot 10^{-2}$, which implies a bitter fit for the nonlinear analysis, which is why this was used for further curve-fitting.

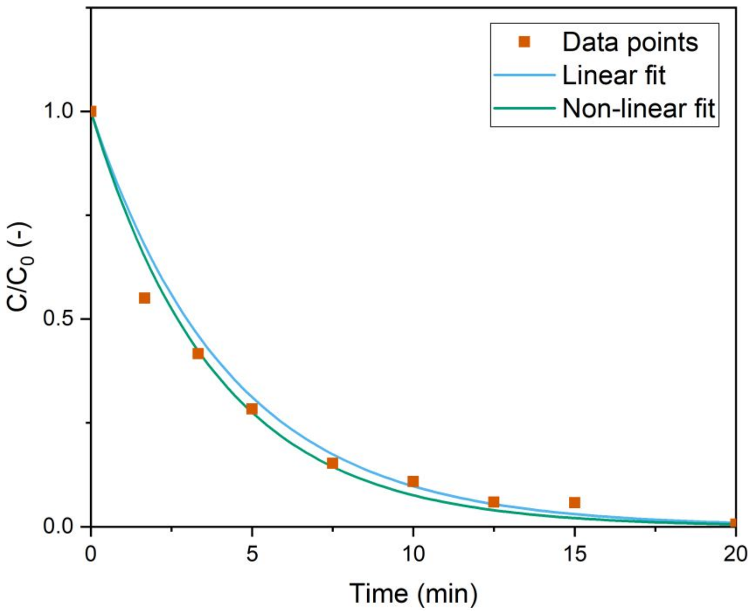

Figure S7. Linear and non-linear fit of first order reaction of the MB degradation in batch. Light power input 60 W (13 W/mL), 10 ppm MB, $C_{TiO_2} = 1.0$ mg/mL, 4.0 mL/min O_2 bubbling.

To ensure that MB adsorption did not affect the reproducibility of the results, batch experiments were performed with and without 30 minutes of pre-mixing (**Figure S8**). No significant difference was found between the performed experiments, implying that the adsorption equilibrium is established fast enough to allow for experiments without the need for pre-mixing.

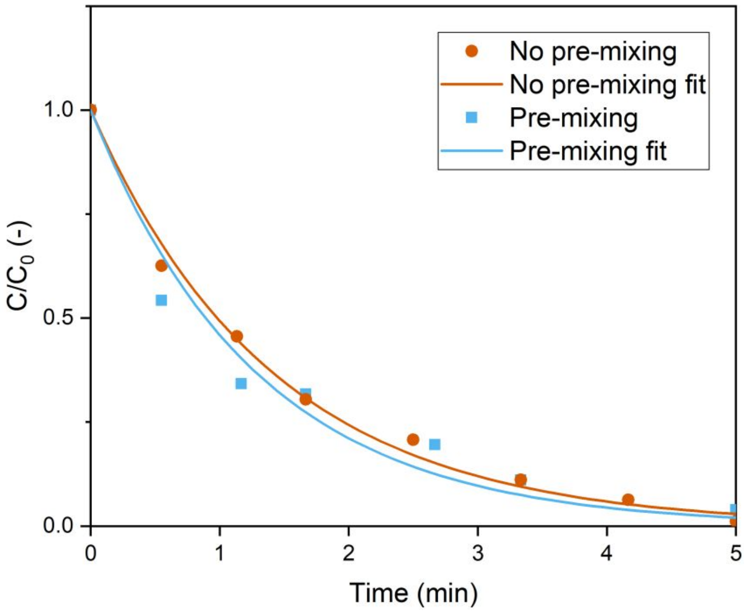

Figure S8. Batch experiments with and without 30 minutes of pre-mixing of the sample. Light power input 60 W (13 W/mL), 10 ppm MB, $C_{TiO_2} = 1.0$ mg/mL, 4.0 mL/min O_2 bubbling.

For the variation of the light power input, the found behavior was not linear, which can be explained when looking at the relevant kinetic and mass transfer steps. The mass transfer of O_2 to the bulk liquid, mass transfer of the MB and O_2 to the catalyst surface and the reaction event occurring on the catalyst surface all contribute to the overall rate constant (**Eq. S3**). Only the kinetic reaction rate constant, and not the mass transfer steps, should be affected by the light power input (where the light power output is linear to the power input), so

plotting the reciprocal of the overall reaction rate constant versus the reciprocal of the light input power should show the linear relation (**Figure S9**).

$$\frac{1}{k} = \frac{1}{k_{kin}(P) \cdot a_{TiO_2}} + \frac{1}{k_{GL} \cdot a_{GL}} + \frac{1}{k_{LS} \cdot a_{TiO_2}} \qquad \textbf{Eq. S3}$$

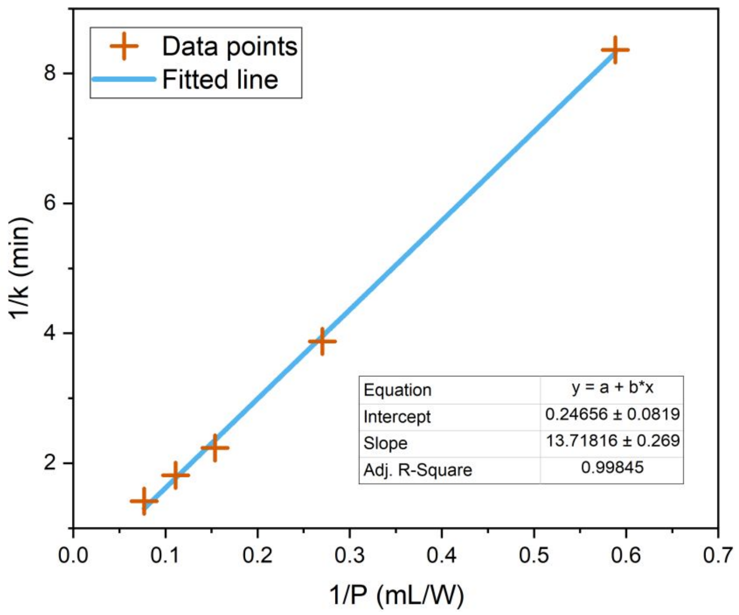

Figure S9. Reciprocal of the apparent reaction rate constant vs. the reciprocal of the light power input. Data obtained with 10 ppm MB, $C_{TiO_2} = 1.0$ mg/mL, 4.0 mL/min O_2 bubbling.

4. Flow results

The effect of the gas-to-liquid ratio (G:L) is shown in **Figure S10** for varying rotation speed. At lower rotation speeds the additional oxygen excess seems to enhance the photodegradation (possibly introducing more mixing into the system by hydrodynamics), but this effect diminishes at higher rpm, where the rotation can provide the additional required mixing for for conversion.

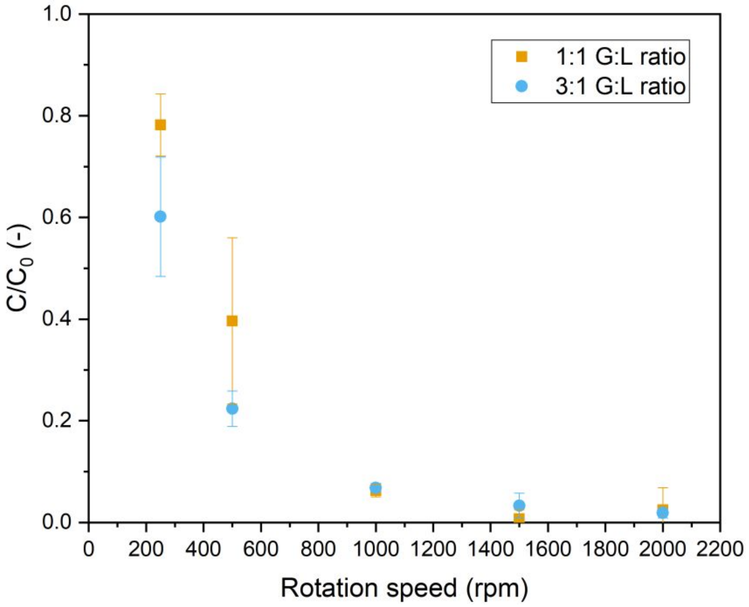

Figure S10. Normalized concentration of methylene blue vs. rotation speed for two different G:L ratios. Light input power 175 W (6.5 W/mL), $\Phi_{V,L} = 13.5$ mL/min, 10 ppm MB, $C_{TiO_2} = 10$ mg/mL.

Determining the overall reaction rate constant for the flow setup was done by approximating the pRS-SDR as a perfect single CSTR. As mentioned in the paper and previously reported,[1] the gas holdup is low even at high G:L ratios. For the liquid residence time, a gas holdup of 0 is assumed. This underestimates the productivity and overall reaction rate constant, because this calculated liquid (suspension) residence time is always higher than the actual experimental value. Using these assumptions, the steady-state molar balance of MB in the pRS-SDR (**Eq. S4**) can be re-arranged to calculate the overall reaction rate constant (**Eq. S5**). Here, C_{MB} is the MB concentration, V_L is the liquid suspension volume of the reactor (27 mL), $\Phi_{V,L}$ is the liquid suspension flow rate (mL/min), τ_L is the liquid residence time (min) and X is the conversion (-).

$$\frac{dC_{MB}V_L}{dt} = \Phi_{V,L}C_{MB,in} - \Phi_L C_{MB} - kC_{MB}V_{V,L} = 0 \quad \textbf{Eq. S4}$$

$$k = \frac{1}{\tau_L}\left(\frac{1}{1-X} - 1\right) with\ \tau_L = \frac{V_L}{\Phi_{V,L}} \quad \textbf{Eq. S5}$$

In **Figure S11** the direct photolysis (no photocatalyst) in the pRS-SDR is shown for varying rotation speeds. As expected, and in agreement with the batch experiments, this effect is negligible. Each point is the avergae of a duplo experiment, with the error bars indicating the 95% confidence interval.

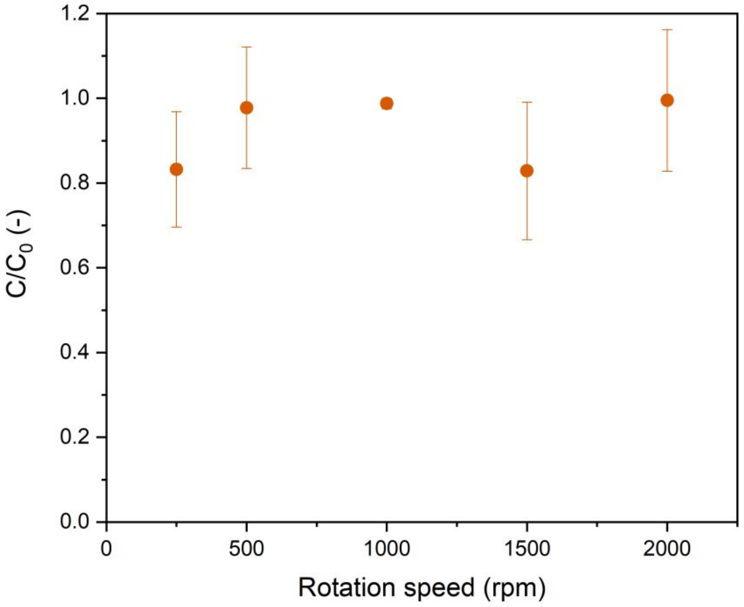

Figure S11. Normalized concentration of MB vs. rotation speed. Light input power 175 W (6.5 W/mL), $\Phi_{V,L} = 13.5$ mL/min, G:L ratio 1:1, 10 ppm MB, $C_{TiO_2} = 0$ mg/mL.

To check the possibility of catalyst build-up in the reactor, the catalyst concentration at the outlet was determined by collecting samples and letting them dry in an oven. **Table S1** shows the difference in measured concentration at the outlet relative to the feed. At low rotation speeds and low catalyst concentration the highest deviation was found. This could be due to solid accumulation at lower rotation speeds. However, at higher rotation speeds and concentrations this is not observed.

Table S1. The catalyst concentration of the outlet mixture for different conditions compared to the catalyst concentration of the feed to check the behavior of the solid in the reactor. The following characterization was carried out for 27 mL min $^{-1}$ with a gas to liquid ratio of 1:1.

Rotation speed (rpm)	Inlet catalyst concentration (mg/mL)	Relative outlet deviation (%)
250	10	-15
500	10	-4.1
1000	10	-5.8
250	45	0.75
500	45	1.8
1000	45	3.1
1500	45	3.5
2000	45	1.3

5. High Speed Imaging of the pRS-SDR

To obtain a better understanding of the hydrodynamics of the reactor, imaging of the system was carried out with a high-speed camera (AOS L-PRI 1000) with a macro lens (Canon EFS 60 mm) in combination with a high intensity LED based light source (Veritas Constellation 120E15). For a certain selected condition, first the reactor was allowed to reach steady state before imaging was initiated. The camera was mounted at $3.7 \cdot 10^{-1}$ m from the top of the quartz plate and images were taken at 980 frames per second. **Figure S12** illustrates the setup used.

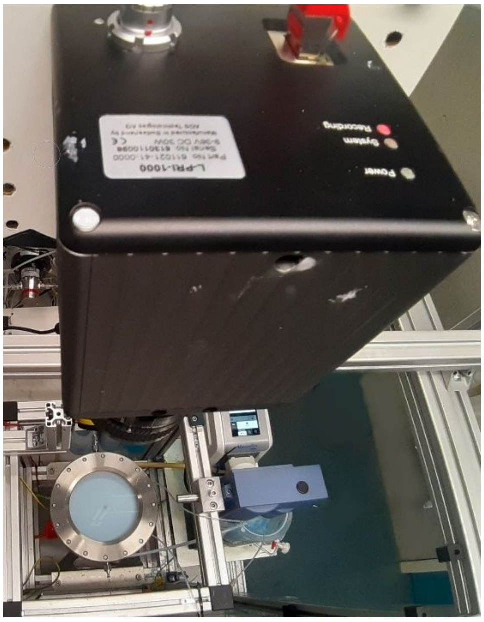

Figure S12. A view of the setup used for imaging

The first images were taken of a gas-liquid system. We have previously carried out image analysis of a gas-liquid pRS-SDR in our previous publication,[1] however, for both completeness and for a comparison to a gas-liquid-solid system, we have redone some imaging with the experimental conditions of this work.

A

B

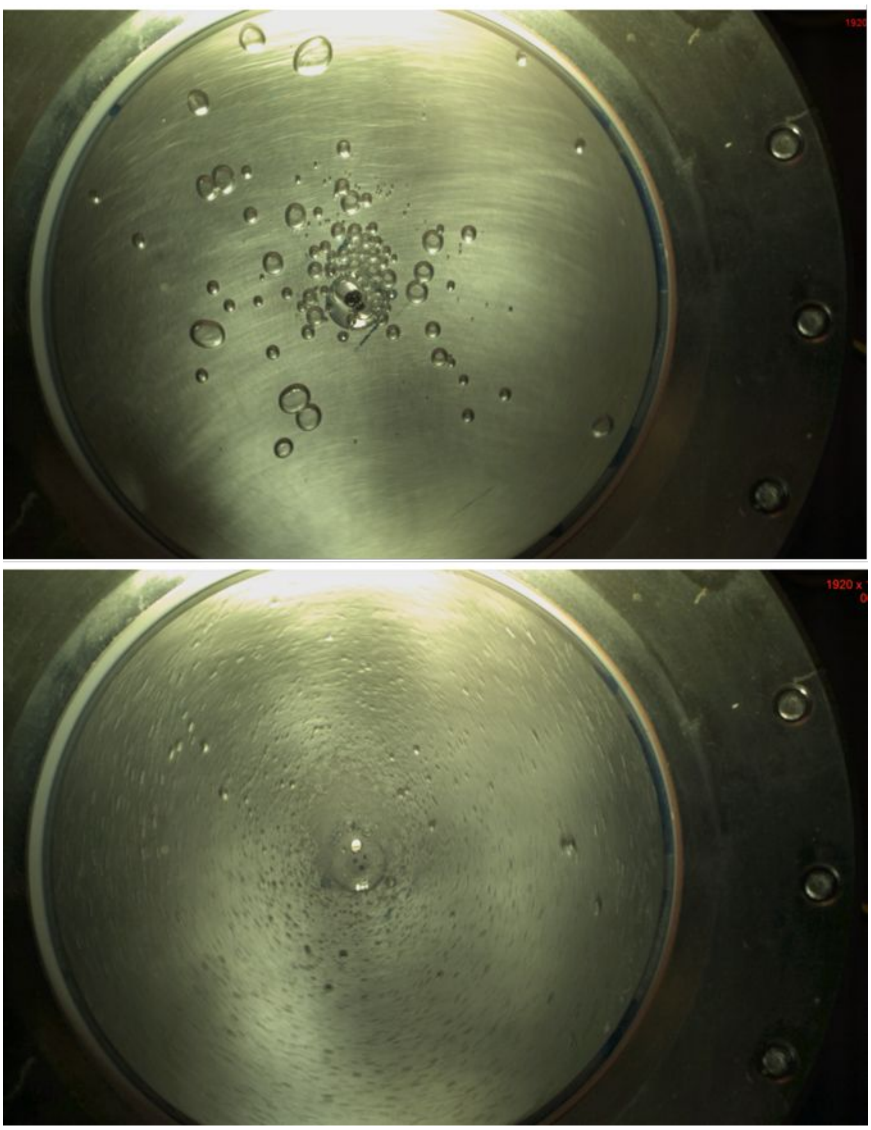

Figure S13. Images of the pRS-SDR at A) 250 rpm and B) 1000 rpm for a gas-liquid system. The gas and liquid flow ratio was 1:1, with a liquid flow rate of 27mL/min.

With the liquid and gas being co-fed from the bottom, we can expect to see a dispersed flow characteristics on the top of the reactor. As can be observed, and as was illustrated in our previous work, the gas bubbles are rather large at 250 rpm (**Figure S13**). However, with increasing rotation speed, due to the high amount of shear present in the reactor, the gas bubbles decrease in size and become much more well dispersed in the system.

In the next set of images, solid was introduced into the reactor as a slurry (1 mg/mL) using the same method as described in the main text (**Figure S14**). The solution begins to appear opaque due to the presence of solid particles. However, the gas bubbles have similar characteristics (large) at 250 rpm. At higher rotation speeds, the gas bubbles presumably again become dispersed into the solution, but unlike in the case of no solids, due to the opacity, it becomes difficult to observe these smaller bubbles. A slight amount of MB was added to the inlet solution to increase the contrast; however, that still did not help to image the smaller bubbles at 2000 rpm. Similarly, not much can be characterized about the slurry. The solution becomes completely opaque and with 1 mg/mL, not much can be observed regarding the particles in solution.

A

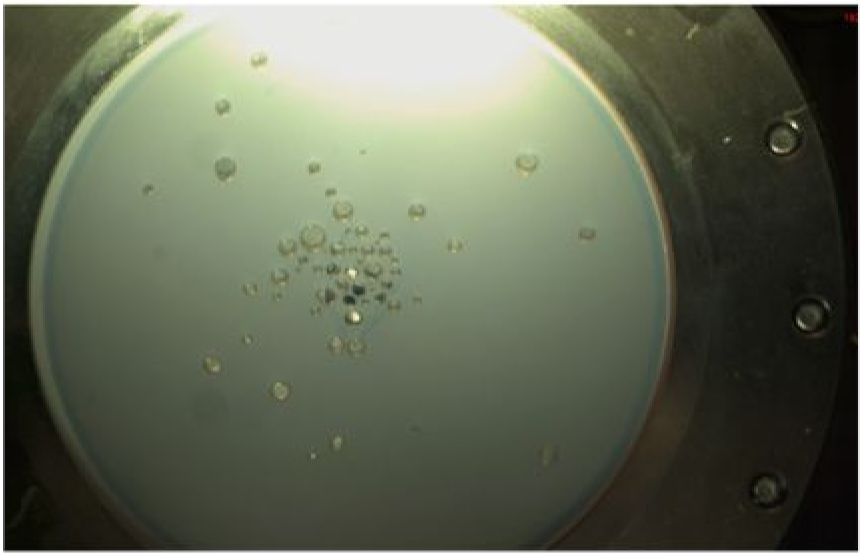

B

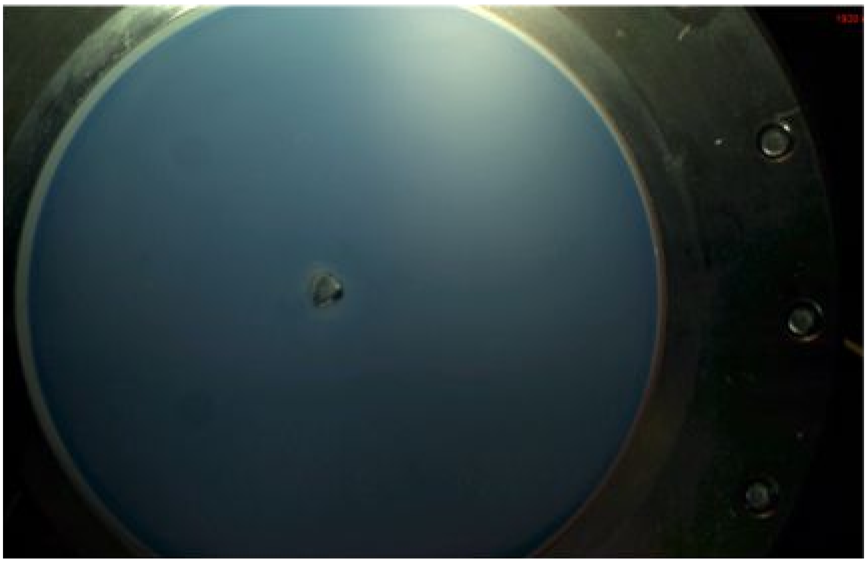

Figure S14. Images of the pRS-SDR at A) 250 rpm and B) 2000 rpm for a gas-liquid-solid system. The gas and liquid flow ratio was 1:1, with a liquid flow rate of 27 mL/min. The slurry concentration in the liquid flow was 1 mg/mL.

As the slurry concentration is increased to 10 mg/mL in the feed, we did begin to observe what was most likely some particle aggregation at low rotation speeds. This has been highlighted using red circles in **Figure S15**. However, the presence of gas bubbles as well as solid particles makes it difficult to distinguish between the two. Therefore, we have only carried out a qualitative assessment of the mixing here. At higher rpms, neither the bubbles nor the particles are observable, indicating much better mixing characteristics.

A

B

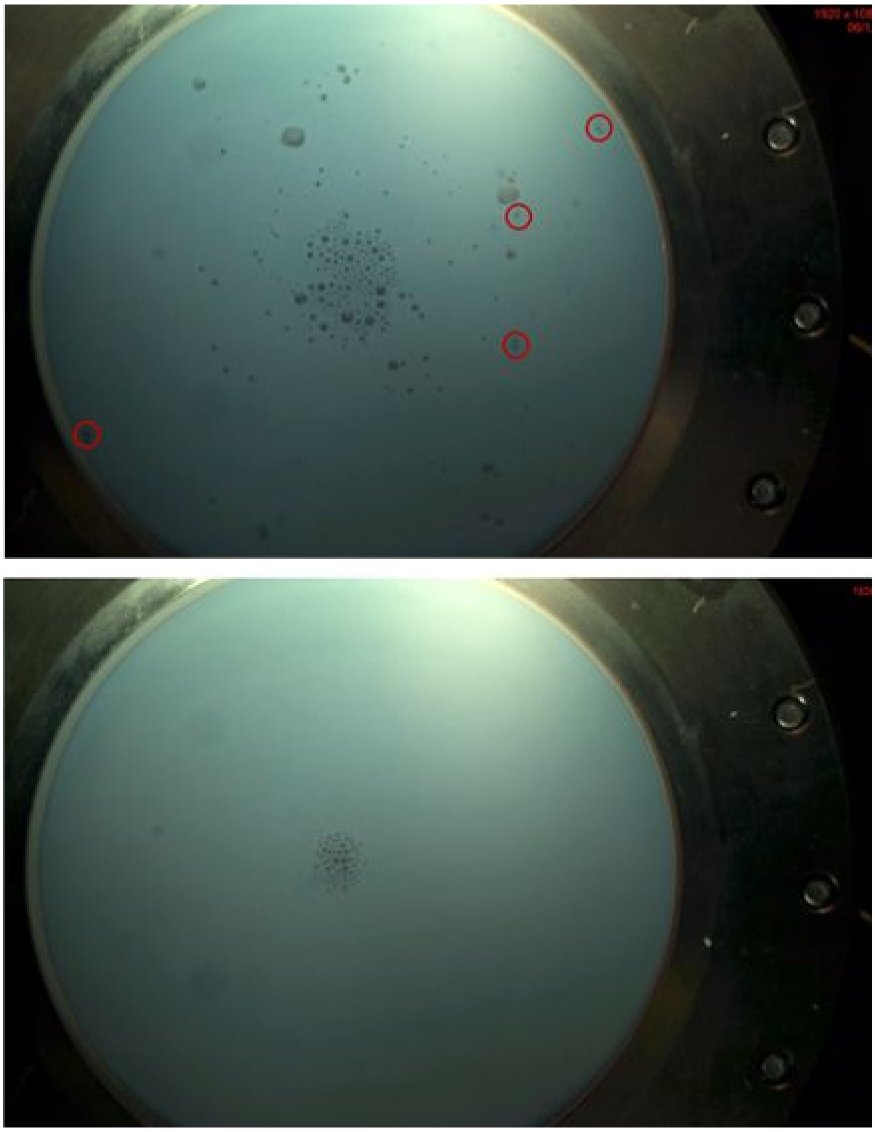

Figure S15. Images of the pRS-SDR at A) 500 rpm and B) 2000 rpm for a gas-liquid-solid system. The gas and liquid flow ratio was 1:1, with a liquid flow rate of 27 mL/min. The slurry concentration in the liquid flow was 10 mg/mL. The red circles in the top pictures identify what appears to be particle agglomeration.

6. References

[1] Chaudhuri, A.; Kuijpers, K. P. L. L.; Hendrix, R. B. J. J.; Shivaprasad, P.; Hacking, J. A.; Emanuelsson, E. A. C. C.; Noël, T.; van der Schaaf, J. Process Intensification of a Photochemical Oxidation Reaction Using a Rotor-Stator Spinning Disk Reactor: A Strategy for Scale Up. *Chem. Eng. J.*, **2020**, *400*, 125875.
